# Src activation in the hypothalamic arcuate nucleus may play an important role in pain hypersensitivity

**DOI:** 10.1038/s41598-019-40572-z

**Published:** 2019-03-07

**Authors:** Hanpei Ma, Chunxu Yao, Peng Ma, Ju Zhou, Shan Gong, Jin Tao, Xian-Min Yu, Xinghong Jiang

**Affiliations:** Key Laboratory of Pain Basic Research and Clinical Therapy, Department of Physiology and Neurobiology, Medical College of Soochow University, Suzhou, 215123 China

## Abstract

Src family of kinases (SFKs) has been found to play an important role in the regulation of nociception. However, how each member of this family acts in the central nervous system (CNS) structures involved in the relay and/or modulation of nociceptive signals, and thereby contributes to the formation and maintenance of pain hypersensitivity, is still a challenge. In this work, a combined study using biochemical, genetic and behavioral approaches was conducted. We found that the expression of activated SFKs in the hypothalamic arcuate nucleus (ARC) area was significantly increased following the development of inflammation induced by injection of complete freund’s adjuvant (CFA) into the hind paw of rats. Furthermore, we identified that Src, but not Fyn or Lyn in the Src family, was activated, and that Src knockdown in the ARC area blocked the inflammation-induced increases in the expression of activated SFKs, the *N*-Methyl-D-aspartate receptor (NMDAR) GluN2B subunit and phosphorylated GluN2B at Y1472 in this region. Moreover, the CFA injection-induced allodynia and hyperalgesia, and the analgesic effect produced by systemic application of the SFK inhibitor, SU6656, were significantly diminished. However, the Src knockdown did not induce any change in the expression of activated SFKs  and the NMDAR GluN2B subunit in normal rats which were not injected with CFA. Neither the Src knockdown nor the systemic application of SU6656 affected the mechanical and thermal sensitivity of the normal rats. Thus, Src activation in the ARC may be a key event for formation and maintenance of pain hypersensitivity associated with peripheral inflammation.

## Introduction

Neuronal sensitization in supraspinal structures of the central nervous system (CNS) triggered by excitation of peripheral nociceptors has been recognized as an important event associated with pain hypersensitivity^1–5^. However, detailed molecular mechanisms underlying the formation and maintenance of the sensitization in supraspinal CNS structures remains largely unknown. Previous studies have shown that neurons in the hypothalamic arcuate nucleus (ARC) area contain β-endophine and play important roles in the descending modulation of nociception^6–8^. Recent studies have demonstrated that with the development of inflammation induced by injection of trinitrobenzene sulfonic acid into the intra-pancreatic duct or complete freund’s adjuvant (CFA) into the hind paw of rats, the activity of Src family of kinases (SFKs) and the tyrosine phosphorylation of *N*-Methyl-D-aspartate receptors (NMDARs) in the ARC area are significantly increased^9–12^. An inhibition of SFKs in this area significantly reduces hyperalgesia and allodynia associated with the chronic pancreatitis or inflammation in the hind paw^9–12^.

Nine members of SFKs are highly expressed in the nervous system. Several members (*e*.*g*. Src, Fyn and Lyn) of SFKs have been found to be involved in the regulation of nociceptive functions^2,13–19^. It is known that each member of SFKs shares a common topology^14,20–22^: the catalytic (SH1) domain is located near the C-terminus and contains an activation loop with an important tyrosine residue (*e*.*g*. Y416 in chicken cellular Src). The N-terminus of the catalytic domain links to an SH2 domain followed by an SH3 domain. The N-terminus (SH4 domain) of each member is involved in membrane association. Only the region between the SH3 and SH4 domains is unique in each member of SFKs^14,20–22^. When SFKs are activated, the tyrosine residue (*e*.*g*. Y416 in chicken cellular Src) in the activation loop of SFKs is autophosphorylated^14,20–22^.

Each member of SFKs may play distinct roles in specific signaling pathways and cellular functions while the considerable redundancy of these kinases has also been noted in the functions, both with respect to the signaling pathways that activate these kinases and the downstream effectors that mediate their biological activities^14,20–22^. Thus, understanding how each member of SFKs acts in every CNS structures involved in the relay and/or modulation of pain signals will be crucial for understanding a scientific basis for developing new therapeutic approaches to treat and/or prevent the development of pain from acute to chronic states. Since the SFK members Src, Fyn and Lyn have been found to be involved in the regulation of nociceptive functions in the spinal cord^13–19^, in this work we examined a hypothesis that all of these kinases in the ARC area might also be involved in the regulation of nociceptive functions.

## Results

### Src, but not Fyn or Lyn, in the ARC area was activated following the development of peripheral inflammation induced by the injection of CFA into the hind paw

**Figure 1:**
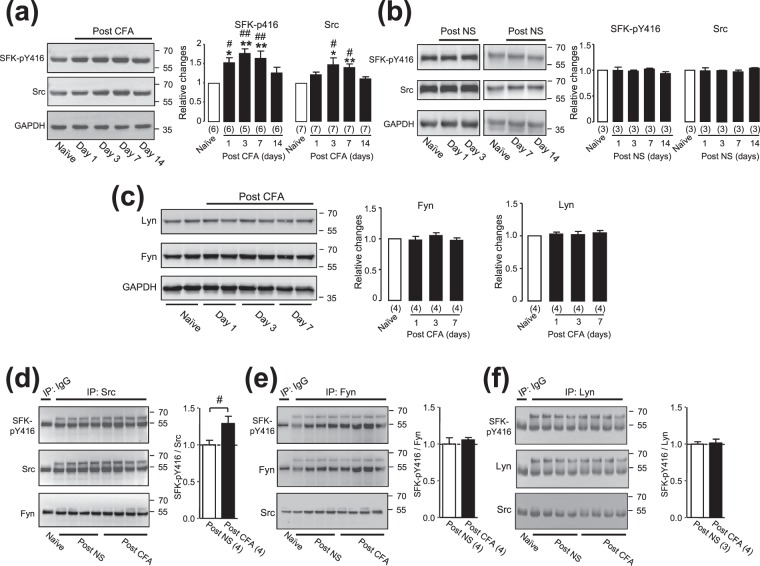
The expression of phosphorylated SFKs at Y416, Src, Fyn or Lyn in the ARC area following the CFA injection into the hind paw of rats. Gels shown in (**a**–**c**) were loaded with lysates prepared from the hypothalamic ARC region of animals without any treatment (Naïve) or at days 1, 3, 7 and 14 after the CFA (**a**,**c**) or normal saline [NS, (**b**)] injection into the hind paw as indicated. Each group of blots was cropped from a same filter (For an example see Fig. S2), stripped and successively probed with antibodies as indicated on the left of blots. Values on the right side of blots indicate the molecular mass (Kd). The ratio of band intensities versus that of GAPDH was calculated and then normalized to the ratio in naïve animals (=1, open bar) for determining relative changes. Bar graphs show summary data (mean ± SEM) of the relative changes. *, **p < 0.05, *p* < 0.01, Dunnett’s post hoc test in one-way ANOVA in comparisons with those of naïve rats. ^#,##^*p* < 0.05, *p* < 0.01, Bonferroni’s post hoc test in two-way ANOVA in comparison between rats which received CFA versus saline injections into the hind pawl. Gels (from left to right) shown in (**d**–**f**) were loaded with immunoprecipitates performed using non-selective IgG from ARC tissues of naïve rats in the same experimental sets, or using antibodies recognizing Src, Fyn or Lyn from ARC tissues of rats at day 3 after the injection of normal saline or CFA into the hind pawl. The group of blots shown in (**d**) or (**e**) was cropped from a same filter, and the group of blots shown in (**f**) was from the full length filter shown in Fig. S5. Each group of blots was stripped and successively probed with antibodies as indicated: the SFK-pY416 antibody for top blots in (**d**–**f**); the Src, Fyn and Lyn antibodies (which were used to precipitate Src, Fyn and Lyn, respectively) were used to reprobe the filters shown in middle blots in (**d**–**f**), respectively; the Fyn antibody was used to probe the filter shown in the bottom blot in (**d**) and the Src antibody to probe the filter shown in the bottom blots in (**e**,**f**). Bands detected in the immunoprecipitates of non-selective IgG indicate positions of heavy chains of the antibodies used. The original blots of those displayed in (**d**–**f**) are shown in Figs S3–S5, respectively. The ratios of the band intensities of SFK-pY416 versus that of Src, Fyn and Lyn immunoprecipitated were respectively calculated. Bar graphs show summary data. NS: rats which received the injection of normal saline into the hind paw. CFA: rats which received the CFA injection into the hind paw. ^#^*p* = 0.038, t_6_ = 2.64, unpaired *t*-test in the comparison of rats receiving the injection of normal saline versus ACF into the hind paw. Values in brackets indicate the number of experimental repeats.

In order to determine whether SFKs in the ARC area may be activated following the development of peripheral inflammation, an antibody which recognizes the phosphorylated tyrosine residue in the action loop of SFKs (e.g. Y416 in chicken cellular Src) was used in this work. Since this antibody may detect phosphorylated Src, Fyn and Lyn at sites equivalent to Y416 (see manufacturer’s documents at https://www.cellsignal.com), it was labeled as SFK-pY416 antibody in the figures and following text. When compared with that detected in rats which received no treatment (naïve), the expression of phosphorylated SFKs at Y416 in the ARC area was significantly increased at day 1 after the CFA injection into the hind pawl of rats (*p* < 0.05, Dunnett’s post hoc test in one-way ANOVA; Fig. 1a). This increase lasted for 7 days (Fig. 1a). Src expression in this area was significantly increased at days 3 and 7 after the CFA injection (*p* < 0.01 for day 3 and *p* < 0.05 for day 7; Fig. 1a). No significant increase in the expression of SFK-pY416 or Src could be found in the ARC area of rats after the injection of normal saline (NS) into their hind paws (*p* > 0.05, Dunnett’s post hoc test in one-way ANOVA; Fig. 1b). The comparisons between rats which received injections of CFA versus NS into the hind pawl indicated that the expressions of SFKs phosphorylated at Y416 and protein Src in the ARC area were significantly increased after the CFA injection (SFK-pY416: *p* < 0.05 at day 1, *p* < 0.01 at days 3 and 7; Src: *p* < 0.05 at days 3 and 7 after the CFA injection; Bonferroni post hoc test in two-way ANOVA; see Fig. 1a,b). The expression of Fyn or Lyn in the ARC area appeared to be similar to that in naïve rats (*p* > 0.05, Dunnett’s post hoc test in one-way ANOVA; Fig. 1c). Since the SFK-pY416 antibody may detect phosphorylated Src, Fyn and Lyn at sites equivalent to Y416, we then examined the phosphorylation of immunoprecipitated Src, Fyn or Lyn at the site Y416 from the ARC area at day 3 after the CFA injection (Fig. 1d–f). Compared with those of rats after the saline injection into the hind paw, the ratio of phosphorylated Src detected with a SFK-pY416 antibody versus total Src immunoprecipitated in rats after the CFA injection was significantly increased (*p* = 0.038, t_6_ = 2.64, unpaired *t*-test; Fig. 1d) while no such increase was found in Fyn (*p* = 0.67, t_6_ = 0.45; Fig. 1e) or Lyn (*p* = 0.79, t_5_ = 0.28; Fig. 1f). No Fyn was found in proteins immunoprecipitated with a Src antibody (see Fig. 1d) and no Src was found in proteins immunoprecipitated with a Fyn or Lyn antibody (see Fig. 1e,f).

**Figure 2:**
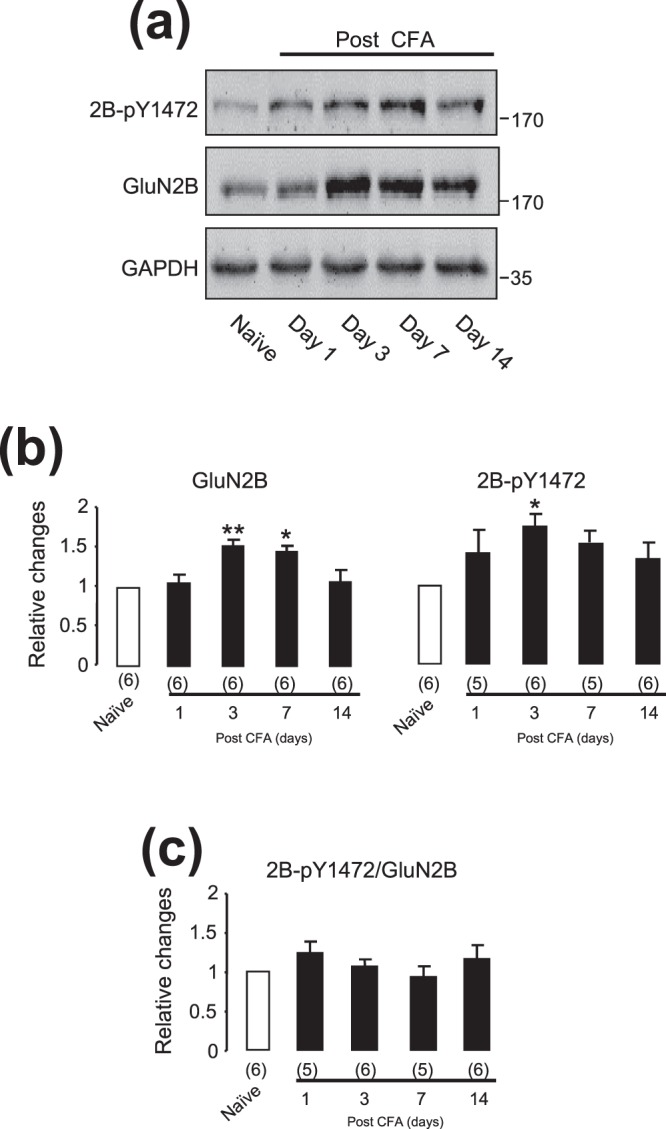
The expression of GluN2B or phosphorylated GluN2B at Y1472 in the ARC area following the CFA injection into the hind paw of rats. The gel shown in (**a**) was loaded with lysates prepared from the hypothalamic ARC area of animals without any treatment (Naïve) or at days 1, 3, 7 and 14 after the CFA injection into the hind paw. The group of blots were cropped from a same filter, and stripped and successively probed with an antibody against GluN2B-pY1472 (2B-pY1472, top blot), GluN2B (middle blot) or GAPDH (bottom blot). The ratio of the band intensity of GluN2B-pY1472 or GluN2B versus that of GAPDH was calculated. Bar graphs in (**b**) show normalized ratios to those in naïve animals ( = 1). The bar graph in (**c**) shows the ratios of the band intensities of GluN2B-pY1472 versus GluN2B. *, **p < 0.05, *p* < 0.01, Dunnett’s post hoc test in one-way ANOVA in comparison with those of naïve rats. Values in brackets indicate the number of experimental repeats.

When compared with those in naïve animals, the expressions of both the NMDAR GluN2B (*p* < 0.01 for day 3 and *p* < 0.05 for day 7, Dunnett’s post hoc test in one-way ANOVA) and the phosphorylated GluN2B at Y1472 (*p* < 0.05 for day 3) in the ARC area were increased after the CFA injection (Fig. 2a,b). However, the ratio of the phosphorylated versus total GluN2B was not significantly changed (*p* > 0.05, Dunnett’s post hoc test in one-way ANOVA; Fig. 2c).

### Src knockdown in the ARC area blocked the CFA injection-induced increase in the expression of phosphorylated SFKs at Y416

**Figure 3:**
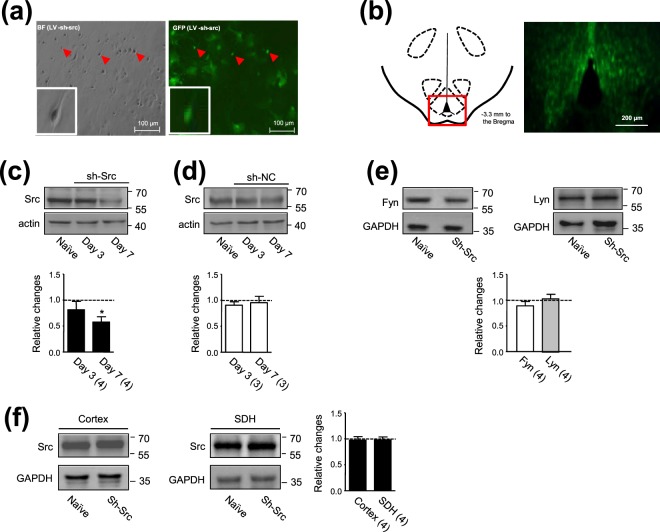
The infection of sh-Src in the ARC area reduced Src expression in this area. Images (left and right) in (**a**) recorded from the same field of cultured ARC neurons show lentivirus-mediated GFP and sh-Src infection; Bar: 100 µm. Arrowheads indicate infected neurons. A standard diagram in (**b**) (left) indicates the coronal section at 3.3 mm posterior to Bregma^39^. A fluorescence image in (**b**) (right) was recorded from the ARC area indicated within the red square on the diagram (left). The NeuN antibody co-labeling of ARC neurons is shown in Fig. S1. Gels shown in (**c**) and (**d**) were respectively loaded with lysates prepared from the ARC area of naïve animals, and animals at days 3 and 7 after the infusion of sh-Src (**c**) or sh-NC (**d**) into the ARC area. Gels shown in (**e**) were respectively loaded with lysates prepared from the ARC area of naïve animals and animals at day 7 after intra-ARC infusion of sh-Src. Gels shown in (**f**) were respectively loaded with lysates prepared from the cerebral cortex (left blots) and the dorsal horn of the lumbar spinal cord (SDH, right blots) of naïve animals and animals at day 7 after the intra-ARC infusion of sh-Src. Each group of blots was cropped from a same filter, and probed with antibodies as indicated on the left side of blots. An example demonstrating the probe of a full length filter is shown in Fig. S6. The ratio of the band intensity of Src, Fyn or Lyn versus that of actin or GAPDH was calculated. Bar graphs in (**c**–**f**) respectively show the summary data (mean ± SEM) of ratios normalized to those in naïve animals (=1, dashed line). *p < 0.05, Dunnett’s post hoc test in one-way ANOVA in comparison with that of naïve rats. Values in brackets indicate the number of experimental repeats.

**Figure 4:**
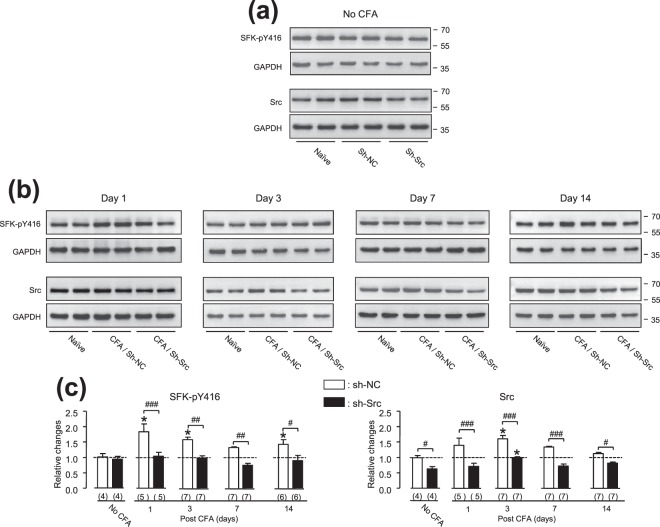
Src knockdown in the ARC area prevented the CFA injection-induced increases in the expression of Src and phosphorylated SFKs at Y416 in this area. The gel shown in (**a**) (from left to right) was loaded with lysates respectively prepared from ARC tissues of rats without any treatment (Naïve, left) and rats which received the intra-ARC infusion of sh-NC (sh-NC, middle) or sh-Src (sh-Src, right) but no CFA injection (No CFA). Lysates prepared from ARC tissues of rats (which received the intra-ARC infusion of sh-NC or sh-Src 7 days before) at days 1, 3, 7 and 14 after the CFA injection into the hind paw were respectively loaded in the middle (CFA/sh-NC) and right (CFA/sh-Src) two lanes of the gels shown in (**b**). Lysates prepared from ARC tissues of rats in the same experimental sets without any treatment (Naïve) were loaded in the left two lanes of the gels. Sh-NC: rats which received the intra-ARC infusion of sh-NC; Sh-Src: rats which received the intra-ARC infusion of sh-Src. Each group of blots was from a same filter, and stripped and successively probed with SFK-pY416 and GAPDH antibodies or Src and GAPDH antibodies as indicated. The original full length blots are shown in Fig. S7. The ratio of the band intensities versus that of GAPDH was calculated and then normalized to the ratio in naïve animals [ = 1, dashed line in (**c**)] for examining relative changes. Bar graphs show summary data (mean ± SEM) of relative changes. *p < 0.05, Dunnett’s post hoc test in one-way ANOVA in comparison with that of rats which did not receive the CFA injection (No CFA). ^#,##,###^*p* < 0.05, <0.01, <0.001 Bonferroni’s post hoc test in two-way ANOVA in comparison between rats which received sh-Src versus sh-NC infusions in the ARC area; Values in brackets indicate the number of experimental repeats.

Based on the findings that the expressions of Src as well as activated Src in the ARC area were increased following the injection of CFA into the hind paw, we then investigated the effect of infusion of lentivirus particles conjugated with Src shRNA 5′-GCGGCTGCAGATTGTCAATAA-3′ (sh-Src)^23^ into the ARC area. Lentivirus-containing empty vector (sh-NC) was infused as a control (Fig. 3). We found that in cultured ARC neurons, the infection rate of the lentivirus particles could reach 76% of the cells (Fig. 3a). Infections of the ARC area by sh-Src *in vivo* were shown in Fig. 3b. Figure S1 shows infected ARC neurons detected with NeuN antibody co-labeling. When compared with those in rats which did not receive any treatment (naïve), the expression of Src in the ARC area was respectively reduced to 80 ± 16% (n = 4 rats) and 57 ± 10% (n = 4 rats) at days 3 and 7 after the infusion of the virus containing sh-Src [0.5 μL, the titer: 1 × 10^9^ plaque forming unit (PFU)/ml]. Since statistically significant decreases in Src expression in the ARC area occurred in 7 days after the intra-ARC infusion of sh-Src (*p* < 0.05, Dunnett’s post hoc test in one-way ANOVA, Fig. 3c), we then examined effects after 7 days following the virus infusion. The same amount of lentivirus containing empty vector (sh-NC) was examined as a control. The intra-ARC infusion of sh-NC did not produce any change in Src expression (*p* > 0.05, Fig. 3d). The intra-ARC infusion of sh-Src did not induce any significant change in the expression of Fyn or Lyn in the ARC area (*p* > 0.05, Dunnett’s post hoc test in one-way ANOVA, Fig. 3e), or Src in either the cerebral cortex or the dorsal horn of the lumbar spinal cord (*p* > 0.05, see Fig. 3f), indicating that the lentivirus-mediated sh-Src infection in the ARC area was less likely to affect remote CNS areas such as the cerebral cortex and the dorsal horn of the lumbar spinal cord. Furthermore, we found that the expression of phosphorylated SFKs at Y416 was not affected by the intra-ARC infusion of sh-Src either (*p* > 0.05, Dunnett’s post hoc test in one-way ANOVA; Fig. 4a,c) under normal conditions.

The Src knockdown by sh-Src significantly attenuated the CFA injection-induced increases in both phosphorylated SFKs at Y416 and Src in the ARC area (For SFK-pY416: *p*_(interaction)_ = 0.0043, F_4,48_ = 4.36; *p*_(group factor)_< 0.0001, F_1,48_ = 54.55; For Src: *p*_(interaction)_ = 0.342, F_4,55_ = 1.15; *p*_(group factor)_ < 0.0001, F_1,55_ = 54.55; two-way ANOVA; see Figs 1a and 4c). In contrast, the sh-NC infusion did not alter the effect of the CFA injection (For SFK-pY416: *p*_(interaction)_ = 0.108, F_4,48_ = 2.01; *p*_(group factor)_ = 0.7577, F_1,48_ = 0.1; For Src: *p*_(interaction)_ = 0.5576, F_4,55_ = 0.76; *p*_(group factor)_ = 0.547, F_1,55_ = 1.37; two-way ANOVA; see Figs 1a and 4c). Sh-NC infused rats did not show elevated SFK-pY416 and Src at day 7 after the CFA injection, which is different from those found in animals which received the CFA injection but no intra-ARC infusion (see Fig. 1a).

Significant differences were found in the effects of the intra-ARC infusions of sh-Src versus sh-NC (For SFK-pY416: *p*_(interaction)_ = 0.1604, F_4,48_ = 1.72; *p*_(group factor)_ < 0.0001, F_1,48_ = 41.07; For Src: *p*_(interaction)_ = 0.1856, F_4,50_ = 1.61; *p*_(group factor)_ < 0.0001, F_1,50_ = 77.97; two-way ANOVA, see Fig. 4c). When compared with those in rats which received NS injection into the hind paw, the CFA injection into the hind paw induced significant increases in the expression of SFKs phosphorylated at Y416 and protein Src in the ARC area of rats which received intra-ARC infusion of sh-NC (SFK-pY416: *p* < 0.01 for day 1 and *p* < 0.05 for day 3; Src: *p* < 0.01 for day 3, Bonferroni’s post hoc test following two-way ANOVA, see Figs 1b and 4c), but did not induced such increases in rats which received intra-ARC infusion of sh-Src (see Figs 1b and 4c).

### The Src knockdown prevented the CFA injection-induced increases in the expressions of the NMDAR GluN2B subunit and phosphorylated GluN2B at Y1472 in the ARC area

**Figure 5:**
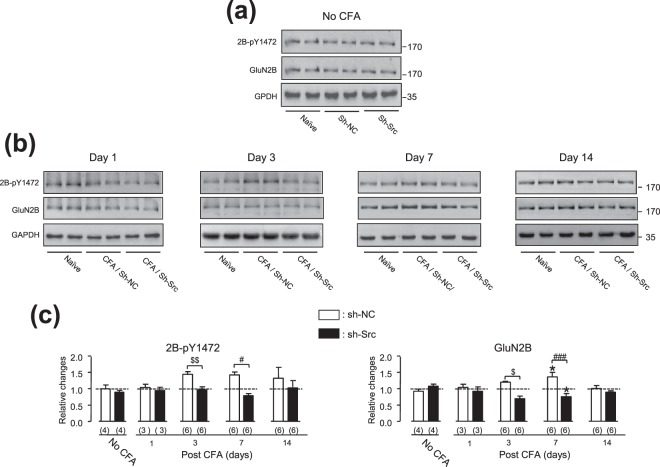
Src knockdown in the ARC area prevented the CFA injection-induced increases in the expression of the GluN2B and GluN2B-pY1472 in this area. The gel shown in (**a**) (from left to right) was loaded with lysates respectively prepared from ARC tissues of rats without any treatment (Naïve, left) and rats which received the intra-ARC infusion of sh-NC (sh-NC, middle) or sh-Src (sh-Src, right) but no CFA injection (No CFA). Lysates prepared from ARC tissues of rats (which received the intra-ARC infusion of sh-NC or sh-Src 7 days before) at day 1, 3, 7 or 14 after the CFA injection into the hind paw were respectively loaded in the middle (CFA/sh-NC) and right (CFA/sh-Src) two lanes of the gels shown in (**b**). Lysates prepared from ARC tissues of rats in the same experimental sets without any treatment (Naïve) were loaded in the left two lanes. Each group of the blots was from a same filter, and stripped and successively probed with an antibody against GluN2B-pY1472 (top blots), GluN2B (middle blots) or GAPDH (bottom blots). The original full length blots are shown in Fig. S8. The ratio of the band intensities versus that of GAPDH was calculated and then normalized to the ratio in naïve animals [=1, dashed line in (**c**)] for examining relative changes. Bar graphs show summary data (mean ± SEM) of relative changes. 2B-pY1472: phosphorylated GluN2B at Y1472; *p < 0.05, Dunnett’s post hoc test in one-way ANOVA in comparison with that of rats which did not receive the CFA injection (No CFA). ^#,###^*p* < 0.05, <0.001 Bonferroni post hoc test in two-way ANOVA in comparison between rats received sh-Src and sh-NC infusions in the ARC area; ^$^*p* = 0.032, *t*_10_ = 2.5; ^$$^*p* = 0.002, *t*_10_ = 4.1, unpaired *t*-test in comparisons between rats received sh-Src and sh-NC infusions in the ARC area. Values in brackets indicate the number of experimental repeats.

In order to determine potential mechanisms underlying the role of Src in the regulation of pain hypersensitivity induced by peripheral inflammation, we investigated the effect of the Src knockdown on the NMDAR GluN2B subunit in the ARC area. We found that the Src knockdown also prevented the CFA injection-induced increases in expressions of the GluN2B and phosphorylated GluN2B at Y1472 in the ARC area (For GluN2B: *p*_(interaction)_ = 0.0002, F_4,40_ = 6.917; *p*_(group factor)_ = 0.0038, F_1,40_ = 9.473; for GluN2B-pY1472: *p*_(interaction)_ = 0.4467, F_4,40_ = 0.9474; *p*_(group factor)_ = 0.0064, F_1,40_ = 8.274; two-way ANOVA, see Fig. 2b and 5c). The sh-NC infusion did not alter the effect of the CFA injection on the expression of GluN2B or phosphorylated GluN2B at Y1472 (For GluN2B: *p*_(interaction)_ = 0.316, F_4,45_ = 1.219; *p*_(group factor)_ = 0.083, F_1,45_ = 3.143; for GluN2B-pY1472: *p*_(interaction)_ = 0.7809, F_4,43_ = 0.4373; *p*_(group factor)_ = 0.141, F_1,43_ = 2.249; two-way ANOVA, see Figs 2b and 5c). The effects of the intra-ARC infusions of sh-Src versus sh-NC were significantly different (For GluN2B: *p*_(interaction)_ = 0.0002, F_4,40_ = 6.917; *p*_(group factor)_ = 0.0038, F_1,40_ = 9.473; for GluN2B-pY1472: *p*_(interaction)_ = 0.4467, F_4,43_ = 0.9474; *p*_(group factor)_ = 0.0064, F_1,43_ = 8.274; two-way ANOVA, see Fig. 5c).

### The CFA injection-induced pain hypersensitivity and the anti-nociceptive effect produced by the systemic application of the SFK inhibitor SU6656 were diminished by Src knockdown in the ARC area

**Figure 6:**
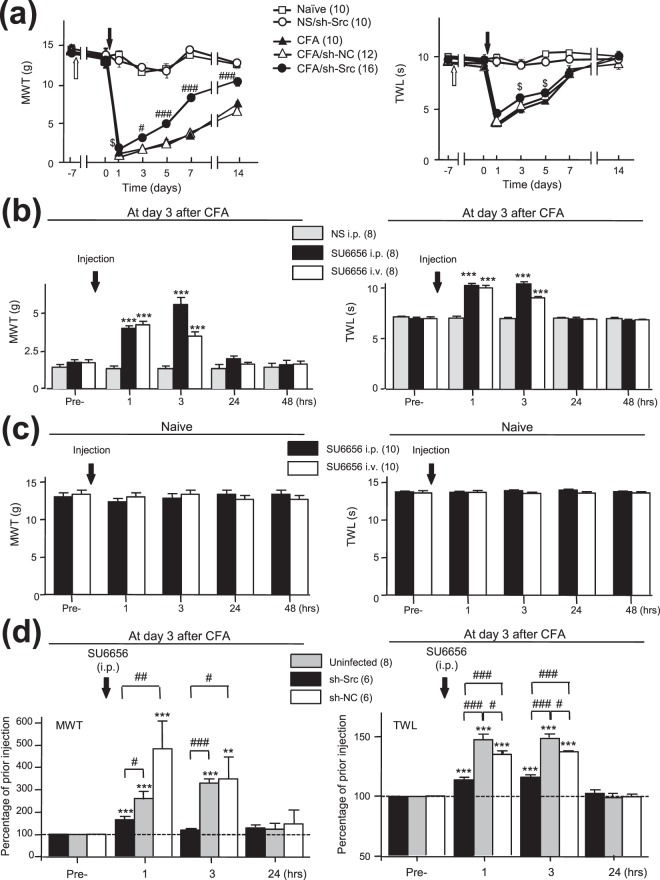
Both the hypersensitivity induced by the CFA injection and the analgesic effect produced by the systemic application of the SFK inhibitor SU6656 were attenuated by Src knockdown in the ARC area. Summary data (mean ± SEM) of the MWT and TWL are shown in (**a**). Naïve (open squares): animals which did not receive any treatment; CFA (filled triangles): CFA was injected into the hind paw of rats which did not receive any intra-ARC infusion; CFA/sh-NC (open triangles): CFA was injected into the hind paw of rats at day 7 after the intra-ARC infusion of sh-NC; CFA/sh-Src (filled circles): CFA was injected into the hind paw at day 7 after the intra-ARC infusion of sh-Src; NS/sh-Src (open circles): normal saline (NS) was injected into the hind paw at day 7 after the intra-ARC infusion of sh-Src; ^#,###^*p* < 0.05, <0.001 Bonferroni’s post hoc test in two-way ANOVA in comparison of the MWT of rats which received the intra-ARC infusion of sh-Src (filled circles) with that of rats which did not receive any intra-ARC infusion (filled triangles). ^$^*p* = 0.016, *t*_24_ = 2.6 for MWT at day 1; ^$^*p* = 0.012, *t*_24_ = 2.7 for TWL at day 3; ^$^*p* = 0.039, *t*_24_ = 2.2 for TWL at day 3; unpaired *t*-test in comparison of the MWT or TWL of rats which received the intra-ARC infusion of sh-Src (filled circles) with those of rats which did not receive any intra-ARC infusion (filled triangles). (**b**) The summary data (mean ± SEM) of the MWT and TWL before and after the administration of SU6656 at day 3 following the CFA injection. i.p.: intraperitoneal injection; i.v.: intravenous injection. (**c**) The MWT and TWL before and after the SU6656 administration in naïve rats. (**d**) The changes in the MWT and TWL relative to those before the administration of SU6656 (=100%, dashed line). Uninfected: rats which did not receive any intra-ARC infusion. Sh-Src: rats which received the intra-ARC infusion of sh-Src; Sh-NC: rats which received the intra-ARC infusion of sh-NC. Pre-: before injection; **, ***p < 0.01, <0.001, Dunnett’s post hoc test in repeated measures one-way ANOVA in comparisons with those before the SU6656 injection. ^#,##,###^*p* < 0.05, <0.01, <0.001 Bonferroni’s post hoc test in repeated measures two-way ANOVA in comparisons between the groups of rats as indicated. Values in brackets indicate the number of rats tested.

To determine the role of Src in the ARC area in the regulation of nociception associated with peripheral inflammation, we investigated the effect of Src knockdown in the ARC area on nociceptive behavior of rats following the CFA injection. Seven days after the intra-ARC infusion of sh-Src, CFA was injected into the hind paw. Prior to the CFA injection the mechanical withdraw threshold (MWT) and thermal withdraw latency (TWL) were 12.9 ± 0.5 g and 9.6 ± 0.4 sec in rats (n = 10) which did not receive any intra-ARC infusion, 13.5 ± 0.3 g and 9.0 ± 0.3 sec in rats (n = 16) which received the intra-ARC infusion of sh-Src, and 15.3 ± 0.6 g and 9.0 ± 0.2 sec in rats (n = 12) which received the intra-ARC infusion of sh-NC, 12.8 ± 0.8 g and 9.6 ± 0.5 sec in naïve rats (n = 10) which received no treatment at the time corresponding to the time point before the CFA injection. No statistically significant difference could be found among these animals (*p* = 0.19, F_3,44_ = 1.65, one-way ANOVA, see Fig. 6a), demonstrating that the intra-ARC infusion of sh-Src or sh-NC did not induce any significant change in either mechanical or thermal sensitivity of rats under normal conditions.

Twenty four hours (day 1) after the CFA injection, the MWT and TWL of rats with no intra-ARC infusion were significantly reduced to 1.3 ± 0.1 g and 3.7 ± 0.4 sec, respectively (*p* < 0.001, Dunnett’s post hoc test in repeated measures one-way ANOVA; Fig. 6a). Such significant reductions in the MWT lasted for 14 days (*p* < 0.001; Fig. 6a) and in the TWL for 5 days after the CFA injection (*p* < 0.001; Fig. 6a).

In animals which received the intra-ARC infusion of sh-Src, the CFA injection-induced decreases in the MWT and TWL were significantly reduced when compared with those in rats which did not receive any intra-ARC infusion (For MWT: *p*_(interaction)_ < 0.0001, F_6,144_ = 11.24; *p*_(group factor)_ = 0.0001, F_1,144_ = 30.39; for TWL: *p*_(interaction)_ = 0.005, F_6,144_ = 3.25; *p*_(group factor)_ = 0.3704, F_1,144_ = 0.83, repeated measures two-way ANOVA, see Fig. 6a). The effect on the MWT could be noted at day 1 (*p* = 0.016, *t*_24_ = 2.60, unpaired *t*-test), which lasted until day 14 after the CFA injection (*p* < 0.05 for day 3, *p* < 0.001 for days 5, 7 and 14; Bonferroni’s post hoc test in repeated measures two-way ANOVA, see Fig. 6a). The effect on TWL could be noted at day 3 (*p* = 0.012, *t*_24_ = 2.7, unpaired *t*-test) and day 5 (*p* = 0.039, *t*_24_ = 2.18) after the CFA injection (Fig. 6a).

The sh-NC infusion did not significantly alter the effect of the CFA injection on the MWT or TWL (For MWT: *p*_(interaction)_ = 0.3589, F_6,120_ = 1.11; *p*_(group factor)_ = 0.0638, F_1,120_ = 3.85; for TWL: *p*_(interaction)_ = 0.3717, F_6,120_ = 1.09; *p*_(group factor)_ = 0.7291 F_1,120_ = 0.12; repeated measures two-way ANOVA, see Fig. 6a). The effects of the intra-ARC infusions of sh-Src versus sh-NC on the MWT were significantly different (For MWT: *p*_(interaction)_ < 0.0001, F_6,156_ = 14.16; *p*_(group factor)_ < 0.0001, F_1,156_ = 52.91; repeated measures two-way ANOVA, see Fig. 6a). Together, these findings have suggested that the knockdown of Src in the ARC area may significantly reduce allodynia and hyperalgesia induced by the CFA injection into the hind paw.

To confirm the role of Src in the ARC area in the facilitation of hypersensitivity associated with peripheral inflammation, we examined whether Src knockdown in the ARC area may affect the effect produced by the systemic inhibition of SFKs. SU6656 (2-oxo-3-(4,5,6,7-tetrahydro-1H-indol-2-ylmethylene)-2,3-dihydro-1H-indole-5-sulfonic acid dimethylamide) is a potent inhibitor of SFKs^24–26^. The IC_50_ of SU6656 *in vitro* for SFK members such as Src, Fyn, Yes and Lyn is less than 0.3 μmol^24^ and Lck at 0.4 μmol^27^. Since the analgesic effect produced by the Src knockdown appeared more effective after 24 hrs following the CFA injection, the effects of intraperitoneal (i.p., 1 μmol/1 ml) or intravenous (i.v., 1 μmol/0.3 ml) administration of SU6656 were examined at 72 hrs (day 3) following the CFA injection. When compared with those before the SU6656 injection both the MWT and TWL at 1–3 hrs after the injection significantly increased (*p* < 0.001, Dunnett’s post hoc test in repeated measures one-way ANOVA, Fig. 6b). As a control, injection of NS (i.p. 1 ml) did not produce such effects (Fig. 6b). No significant change in the mechanical or thermal sensitivity was noted after the SU6656 application in naïve rats (Fig. 6c).

Interestingly, however, the effect of SU6656 on the CFA injection-induced pain hypersensitivity was significantly diminished in rats which received the intra-ARC infusion of sh-Src (For MWT: *p*_(interaction)_ < 0.0001, F_3,36_ = 18.11; *p*_(group factor)_ = 0.0046, F_1,36_ = 12.09; for TWL: *p*_(interaction)_ < 0.0001, F_3,36_ = 38.31; *p*_(group factor)_ = 0.0003, F_1,36_ = 26; repeated measures two-way ANOVA; see Fig. 6d). Relative to those before the SU6656 injection the MWT was increased by 160 ± 37% and 230 ± 31% (n = 8) and the TWL by 48 ± 5% and 50 ± 3% (n = 8) at 1 and 3 hrs after the SU6656 injection into rats which did not receive any intra-ARC infusion (uninfected, Fig. 6d). The MWT was increased by 60 ± 15% and 21 ± 5% (n = 6) and the TWL by 14 ± 2% and 16 ± 2% (n = 6) at 1 and 3 hrs after the SU6656 injection into rats which received the intra-ARC infusion of sh-Src (see Fig. 6d). These increases were significantly less than those in rats which did not receive intra-ARC infusion (MWT: *p* < 0.05 for 1 hr, *p* < 0.001 for 3 hrs after the SU6656 injection; TWL: *p* < 0.001 for 1 and 3 hrs after the SU6656 injection; Bonferroni’s post hoc test in repeated measures two-way ANOVA, Fig. 6d). No such effects on the MWT were found in those of the rats which received the infusion of sh-NC (Fig. 6d). TWLs in rats which received the infusion of sh-NC were increased by 35 ± 3% and 38 ± 1% at 1 and 3 hrs after the SU6656 injection (n = 6, Fig. 6d). Although there were statistically significant reductions in the TWL of rats which received the intra-ARC infusion of sh-NC (*p* < 0.05 for 1 and 3 hrs after the SU6656 injection, Bonferroni’s post hoc test in repeated measures two-way ANOVA, Fig. 6d), the effect of SU6656 on the TWL in rats which received the sh-Src infusion was further significantly attenuated when compared with those of rats which received sh-NC (*p* < 0.001 for 1 and 3 hrs after the SU6656 administration, Fig. 6d).

## Discussion

Consistent with our previous finding^10^, the expression of phosphorylated SFKs at Y416 in the ARC area was significantly increased at day 1 after the CFA injection into the hind pawl of rats, and this increase lasted for 7 days. We found that the expression of Src in the ARC area was increased at days 3 and 7 after the CFA injection. In experiments identifying whether Src, Fyn and/or Lyn may play roles in the CFA injection-induced activation of SFKs, we found that Src, but not Fyn or Lyn, in the ARC area was activated following the CFA injection. Furthermore, we found that Src knockdown in the ARC area blocked the CFA injection-induced increase in the expression of phosphorylated SFKs at Y416. The Src knockdown also prevented the CFA injection-induced increases in the expression of the NMDAR GluN2B subunit and phosphorylated GluN2B at Y1472 in the ARC area.

Previous studies have shown that the application of a GluN2B antagonist or blocking tyrosine phosphorylation of GluN2B at Y1472 in the spinal cord significantly reduces neuropathic pain^15,19^. Our previous studies have documented that the application of a GluN2B antagonist or blocking tyrosine phosphorylation of GluN2B at Y1472 in the ARC area may prevent the up-regulation of neuronal activity in the ARC and pain hypersensitivity induced by the injection of trinitrobenzene sulfonic acid into the intra-pancreatic duct or CFA into the hind paw of rats^9–12^. The present study shows that the expression of activated SFKs in the ARC area was significantly increased at day 1 following the CFA injection while increased expression of Src, NMDAR GluN2B or phosphorylated GluN2B at Y1472 appeared after.

Previous studies showed no significant increase in the expression of GluN2B in the ARC area one week after the CFA injection^10,11^. In this work, we examined the expression of GluN2B at days 1, 3, 7 and 14 following the CFA injection, and found that there were significant increases in the expression of GluN2B at days 3 and 7. Thus, the change in the GluN2B expression following the CFA injection appears to be time-dependent. The knockdown of Src blocked the inflammation-induced activation of SFKs and prevented the increases in the NMDAR GluN2B subunit and phosphorylated GluN2B at Y1472. These findings not only implied that the Src activation may act as an up-stream signal leading to the enhancement of NMDARs in the ARC area, but also revealed a potential pathway which may be involved in the formation and maintenance of the CFA injection-induced pain hypersensitivity.

Many signaling molecules/pathways in multiple CNS structures have been found to be involved in the initiation and maintenance of pain hypersensitivity^1–5,13,15–19^. Detailed investigations have shown that the early phase of pain hypersensitivity induced by the injection of formalin into the hind paw is NMDAR -independent, whereas the later phase is NMDAR-dependent^28,29^. Our present data showed that the expressions of Src, GluN2B and phosphorylated GluN2B at Y1472 were significantly increased at days 3 and 7 after the CFA injection while pain hypersensitivity and the increased expression of phosphorylated SFKs at Y416 occurred at day 1 after the CFA injection. The knockdown of Src in the ARC area appeared to be more powerful for reducing the pain hypersensitivity at later days (days 3, 5, 7, 14 after the CFA injection). It has been shown that breaking the Src interaction with the GluN1 subunit of NMDARs in the spinal cord does not affect the early phase response to the injection of formalin into the hind paw, but significantly reduces the later phase response^30^. Taken together it appears that the Src-GluN2B mechanism in the ARC may play an important role for the maintenance of pain hypersensitivity induced by the CFA injection.

Recent studies have suggested that the systemic application of SFK inhibitors is an effective approach to reduce allodynia and hyperalgesia induced by tissue injury^30–34^. The selectivity of any SFK inhibitor can be dose-dependent^27^. High concentrations of SU6656 (10 μmol) may strongly inhibit other kinases such as AMP-activated protein kinase (AMPK) *in vitro*^27^. Our previous studies in cellular models have demonstrated that 2 μmol of SU6656 down-regulates NMDAR activity in hippocampal neurons and Na^+^-mediated currents in cochlear spiral ganglion neurons through a selective inhibition of SFKs^25,26^. In this work 1 μmol of SU6656 (i.p or i.v.) was applied *in vivo*. The finding that the knockdown of Src in the ARC area significantly reduced the analgesic effect of SU6656, provided an additional line of evidence confirming that the effect of SU6656 observed in this work is very likely through a Src-dependent mechanism. However, detailed issues underlying how Src in the ARC is involved in the analgesic effect of SU6656 applied systemically remain to be clarified.

The CFA injection-induced allodynia and thermal hyperalgesia as well as the analgesic effect produced by the systemic administration of the SFK inhibitor SU6656 were significantly diminished by Src knockdown in the ARC area. In contrast, under normal conditions the Src knockdown did not induce any significant change in the expression of activated SFKs, the GluN2B subunit or the phosphorylated GluN2B at Y1472. Neither the Src knockdown nor the systemic application of the SFK inhibitor SU6656 under normal conditions affected the mechanical and thermal sensitivity. Indeed it was also noted that in comparison with either naïve rats or rats injected with normal saline into the hind pawl sh-NC infused rats did not show elevated expressions of SFK-pY416 and Src at day 7 following the CFA injection into the hind paw, which appeared to be different to those in rats without the intra-ARC infusion. Lentivirus containing empty vectors have been found to induce a relatively limited host-inflammatory response and to produce gene silencing^35^. However, in this work the difference between the CFA/sh-Src and CFA/sh-NC groups is significant. Altogether, these data demonstrated that Src knockdown in the ARC area reduced not only the CFA injection-induced hypersensitivity but also the anti-nociceptive effect produced by the systemic application of the SFK inhibitor SU6656. Thus, Src activation in the ARC may be an important event involved in the facilitation of pain hypersensitivity associated with peripheral inflammation.

We noted that the Src knockdown appeared to be more effective at relieving mechanical allodynia, which has been found to be related to the central sensitization^2,13,36–38^, than thermal hyperalgesia induced by the CFA injection. Mechanism(s) contributing to the distinct effects induced by Src knockdown in the ARC area on mechanical allodynia versus thermal hyperalgesia remain unknown. Indeed, multiple supraspinal structures and signaling pathways have been found to be involved in the modulation of nociceptive signals. While the underlying detailed molecular mechanisms/pathways still need to be clarified, here we have identified a critical role of Src activation in the ARC following peripheral inflammation in the formation and maintenance of pain hypersensitivity.

## Methods

### Animal preparations

All animal experiments were conducted following the guidelines of Animal Care and Use Committee of the Medical College of Soochow University and approved by Ethics Committee of Soochow University in accordance with the ethical standards of the International Association for the Study of Pain (IASP).

Male Sprague-Dawley rats (180–200 g) housed on a 12-hr light/dark cycle with free access to food and water were used. Before any experiment rats were adapted to the testing environment for 3 days. CFA (100 µl) was injected into the hind paw of rats anesthetized with intraperitoneal injection (i.p.) of 4% chloral hydrate (1 ml/100 g). After the CFA injection, rats were allocated in individual cages with food and water, and carefully observed until completely recovered from anesthesia.

### Behavioral test

Rats were placed in an individual transparent chamber on a metal mesh table for 30 min. Then, Von Frey tests were performed as described previously^10,11^. In brief, a series of calibrated Von Frey filaments (Stoelting, Wood Dale, IL), ranging from 0.4 to 26 g, were applied perpendicularly to the plantar surface of rats. Each filament was applied for 5 sec. Paw withdrawal was considered as a positive response. Following the formula: 50% threshold (g) = (10^[Xf+Kδ]^)/10,000 [Xf: the final bending force of the Von Frey filament; K: the tabular value for the pattern of positive/negative responses; δ: 0.22], the pattern of positive and negative responses was converted to the mechanical withdrawal threshold (MWT). Thermal stimulation was conducted through a radiant heat apparatus (Ugo basile, #7360, Italy; I.R. Intensity = 30 mw/cm^2^). Twenty seconds was applied as a cut-off latency to avoid heating-induced tissue damage when the thermal stimulation was gradually increased. The thermal stimulation stopped immediately as soon as the rat lifted his hind paw heated, and the thermal withdrawal latency (TWL) was determined accordingly. All the behavioral tests were conducted by two or three experimenters, only one of whom was not “blind” to the experimental design and condition of the rat under investigation. Each experimenter provided his/her own evaluation. Only evaluations, which were identically defined at least by two experimenters, were taken for the data analysis.

### Lentivirus particle production and intra-ARC delivery

Lentivirus particles conjugated with Src shRNA 5′-GCGGCTGCAGATTGTCAATAA-3′ (sh-Src)^23^ were generated as recommended by the manufacturer (Academia Sinica, Taipei, China). For lentiviral particle production, HEK293T cells were co-transfected with expression vector together with CMV-packing vector containing the cDNA encoding green fluorescent protein (GFP) and the Src shRNA. Seventy two hours after the transfection, supernatants containing lentivirus were collected, ultra centrifuged, and then stored at −80 °C until for use. The titer of the lentivirus was 1 × 10^9^ PFU/ml. Lentivirus-containing empty vector (sh-NC) was used as a control.

For delivery of sh-Src or sh-NC into the ARC area, a guide cannula was implanted as described previously^10,11^. In brief, rats anaesthetized with 4% chloral hydrate (1 ml/100 g, i.p) were placed on a stereotaxic apparatus, and implanted with a sterilized stainless-steel guide cannula (20 gauge), which was positioned at 3 mm dorsal to the ARC (AP: 4.0, L: 0.5, V: 9.8 mm)^39^ and fixed to the skull by dental acrylic. After completing the cannula implantation, a stainless steel stylet was inserted into the implanted cannula to prevent obstruction and clogging. All the rats were closely monitored after the surgery. Penicillin (50000 units, once a day for 3 days) was intramuscularly applied to prevent infection. Stylets were examined and replaced every day.

In order to prevent the spreading of infused lentivirus to a remote area outside the ARC region, only 0.5 µL of the virus was infused into the ARC. In 7 days after the implantation of the guide cannula, when rats were recovered and regained weight to a level similar to that before the surgery, lentivirus (0.5 µL, 1 × 10^9^ PFU/ml) containing sh-Src or sh-NC was slowly (0.5 µL/30 sec) infused into the ARC area through a needle (26 gauge) which was inserted into the guide cannula and extended 3 mm beyond the tip of the guide cannula. Five minutes after completing the infusion, the needle was removed and a stylet was inserted into the guide cannula.

### ARC neuron culture and image observation

ARC neurons in culture were used to examine the lentivirus infection. For ARC neuron culture, a block of ARC tissue was dissected from postnatal rats (1 day old) at 4 °C. Dissociated ARC cells (approximately 10^5^–10^6^ cells/cm^2^) were cultured on glass cover-slips for 1–2 days with Neurobasal^TM^ medium (ThermoFisher, LA, CA) supplemented with GIBCO^®^ B-27^®^ (50x, ThermoFisher) and L-glutamine (0.5 mM, ThermoFisher). The sh-Src or sh-NC (0.5 µL, 10^9^ PFU/ml) was bath applied to cultured ARC cells. Three to five hours after the lentivirus application, cells were washed with 1 × phosphate buffer saline (PBS) and successively cultured. For image observations of cultured neurons infected with sh-Src, the glass coverslips were then mounted with Vectashield® mounting medium (Vecta Labortories, Wexford, PA). Fluorescence images were recorded under an inverted fluorescence microscope (Axio Observer A1, Zeiss, Oberkochen, Germany).

To examine the lentivirus infection in the ARC area *in vivo*, rats which received the intra-ARC infusion of sh-Src or sh-NC, were anaesthetized with 4% chloral hydrate (1 ml/100 g, i.p.), and then perfused transcardially with PBS followed by iced cold paraformaldehyde (4%) in phosphate buffer (0.1 M, pH 7.4). The brain tissue was then sampled and fixed with 4% paraformaldehyde in PBS for 8 hrs, stored in 0.1 M PBS with 30% sucrose at 4 °C for 48 hrs. Coronal sections (15 μm thick) were performed throughout the hypothalamic brain region with cryostat microtome (Leica, CM1900, Nussloch, Germany). The brain slices were then incubated with a NeuN antibody (mouse, 1:250, Merck Millipore, Nanjing, China) overnight. The NeuN antibody staining was visualized by incubation with an Alexa Fluor 555-labeled goat anti-mouse antibody (1:1000; Thermo, Shanghai, China). Fluorescence images were observed under a LSM 700 confocal laserscanning microscope (Carl Zeiss, Gottingen, Germany) and analyzed using NIH ImageJ software (NIH, Bethesda, ML) (see Figs 3b and S1).

### Immunoprecipitation and Western blotting analysis

Immunoprecipitation and Western blotting experiments were performed as described previously^10,12,25,40^. In brief, tissues isolated from the ARC area were homogenized in an ice-cold RIPA buffer (Beyotime Biotechnology, Shanghai, China) supplemented with protease inhibitor cocktail (Selleck Chemicals, Shanghai, China), phosphatase inhibitor cocktail (Selleck Chemicals), PMSF (1 mM, Beyotime Biotechnology) and DTT (0.5 mM, Beyotime Biotechnology). For protein immunoprecipitation the ARC homogenates were washed twice with ice-cold PBS, and subsequently spun at 14000 g for 20 min. The concentration of solubilized proteins was determined by BCA assay (Pierce, Shanghai, China). Solubilized proteins (1 mg) were then incubated with non-selective IgG (1 µg, rabbit, CST, Nanjing, China), or an antibody (1 µg) to Src (rabbit, CST), Fyn (rabbit, Abcam, Suzhou, China) or Lyn (rabbit, CST) at 4 °C for overnight. The immune complexes were collected with 20 μl of protein A/G magnetic beads (Bimake, Shanghai, China) for 2 hrs at 4 °C. Immunoprecipitates were then washed four times with ice-cold PBS, resuspended in 4x loading buffer and boiled for 5 min, subjected to SDS-PAGE.

For Western blotting analysis, after proteins were transferred to 0.45 µm PVDF membranes (Millipore, Suzhou, China), the filters were then repeatedly stripped and successively probed with antibodies (overnight at 4 °C) including SFK-pY416 antibody (rabbit, 1:1000, CST), anti-Src antibody (rabbit, 1:1000, CST), anti-Fyn antibody (rabbit, 1:1000, Abcam), anti-Lyn antibody (rabbit, 1:1000, CST), and an antibody against the GluN2B (rabbit, 1:1000, Novus, Nanjing, China) or phosphorylated GluN2B at Y1472 (rabbit, 1:1000, Novus). Antibody staining bands were visualized through incubation with peroxidase-conjugated goat anti-rabbit IgG (1:8000, MultiSciences, Hangzhou, China) or peroxidase-conjugated goat anti-mouse IgG (1:8000, MultiSciences) for 2 hrs at room temperature. Stripping effects were carefully examined before successive antibody probing. The fact that no specific staining signal could be detected through incubation with a secondary antibody (e.g. peroxidase-conjugated goat anti-rabbit IgG or peroxidase-conjugated goat anti-mouse IgG) without incubation of a primary antibody, confirmed that the stripping was successful. Densitomery analyses of Western blot bands were done with Clinx Image Analysis (Clinx Science Instruments, Shanghai, China). To control variations which may occur in biochemical experiments, samples from naïve animals which did not receive any treatment were always examined in each (or each repeat) biochemical experiment. The probe with anti-actin antibody (mouse, 1:1000, CST) or anti-GAPDH (glyceraldehyde-3-phosphate dehydrogenase, mouse, 1:1000, Goodhere Biotechnology, Hangzhou, China) was conducted as a loading control. The ratio of the band intensity versus that of GAPDH or actin was calculated and then normalized to the ratio in naïve animals for examining relative changes induced by any treatment.

To examine whether the lentivirus infusion in the ARC area may also affect the Src expression in remote CNS tissues, proteins from the cerebral cortex and the dorsal horn of the lumbar spinal cord were sampled and examined. All chemicals used in this work were obtained from Sigma (Shanghai, China) except for those as indicated.

### Data analysis

Multiple tests for examining normality or variance of data were performed to determine which type of statistic tests should be used. Dunnett’s post hoc test in one-way ANOVA, Bonferroni’s post hoc test in two-way ANOVA, unpaired or paired *t*-test was used in this work. All data are expressed as mean ± SEM, *p* < 0.05 was considered statistically significant. All materials, data and associated protocols are available to readers without undue qualifications in material transfer agreements.

## Supplementary information

Supplementary Figures
