# Supplementary material for: Src activation in the hypothalamic arcuate nucleus may play an important role in pain hypersensitivity
Source: Sci Rep. 2019 Mar 7;9:3827. doi: 10.1038/s41598-019-40572-z (PMC6405746; doi:10.1038/s41598-019-40572-z)

Src activation in the hypothalamic arcuate nucleus may play an important role in pain hypersensitivity

Hanpei Ma[§], Chunxu Yao[§], Peng Ma, Ju Zhou, Shan Gong, Jin Tao, Xian-Min Yu[*], Xinghong Jiang[*]

Key Laboratory of Pain Basic Research and Clinical Therapy, Department of Physiology and Neurobiology, Medical College of Soochow University, Suzhou 215123, China

[§]: Contributed equally

* Corresponding authors:

yuxianminyu1981@hotmail.com; jiangxinghong@suda.edu.cn

LEGENDS OF SUPPLEMENTARY FIGURES

Fig. s1. The infection of ARC neurons by lentivirus containing sh-Src sh-Src: the infection of sh-Src in the ARC area (green); NeuN: Neurons detected in the ARC area with a NeuN antibody staining (red). White dashed lines in (**a**) show the border of the third cerebral ventricle as indicated with III. The areas within the white squares on the images in (**a**) are higher magnified and shown in (**b**).

Fig. s2. The Western blot analysis of ARC tissues of rats which received CFA injection. The filter was cropped as indicated with dotted arrows. The top blot was probed with an antibody to SFK-pY416, and the bottom one with a GAPDH antibody. Values on the right side of blots indicate the molecular mass (Kd).

Fig. s3. Original Western blots, parts of which are shown in Fig. 1d The same filter was stripped and successively probed with antibodies to SFK-pY416 (top blot), Src (middle blot) and Fyn (Bottom blot). Values on the right side of blots indicate the molecular mass (Kd). IP: Immunoprecipitation; IgG: non-selective IgG (rabbit).

Fig. s4. Original Western blots, parts of which are shown in Fig. 1e The same filter was stripped and successively probed with antibodies to SFK-pY416 (top blot), Fyn (middle blot) and Src (Bottom blot). Values on the right side of blots indicate the molecular mass (Kd). IP: Immunoprecipitation; IgG: non-selective IgG (rabbit).

Fig. s5. Original Western blots, parts of which are shown in Fig. 1f The same filter was stripped and successively probed with antibodies to SFK-pY416 (top blot), Lyn (middle blot) and Src (Bottom blot). Values on the right side of blots indicate the molecular mass (Kd). IP: Immunoprecipitation; IgG: non-selective IgG (rabbit).

Fig. s6. The Western blot analysis of lumbar spinal dorsal horn (SDH) tissues of rats which received no (naïve) or intra-ARC infusion of sh-Src. The same filter was stripped and successively probed with antibodies to GAPDH (top blot) and Src (bottom blot). Values on the right side of blots indicate the molecular mass (Kd).

Fig. s7. Original Western blots, parts of which are shown in Fig. 4 The gels shown in **(a)** and **(f)** (from left to right) were loaded with the prestained protein ladder (Thermo Scientific) and lysates respectively prepared from ARC tissues of rats without any treatment (Naïve) and rats which received the intra-ARC infusion of sh-NC (sh-NC) or sh-Src (sh-Src) but no CFA injection (No CFA). The blots were stripped and successively probed with an antibody against SFK-pY416 (top blot) or GAPDH (bottom blot). The gels shown in **(b)** and **(g), (c)** and **(h), (d)** and **(i), (e)** and **(j)** (from left to right) were loaded with the prestained protein ladder (Thermo Scientific) and lysates respectively prepared from ARC tissues of naïve

rats without any treatment in the same experimental sets and rats [which received the intra-ARC infusion of sh-NC (CFA/sh-NC) or sh-Src (CFA/sh-Src/) 7 days before] at day 1 to day 14 after the CFA injection as indicated. The blots were stripped and successively probed with an antibody against SFK-pY416 (top blot) or GAPDH (bottom blot) in **(b)** – **(d)**, or an antibody against Src (top blot) or GAPDH (bottom blot) in **(g)** – **(j).** Values on the right side of blots shown in **(e)** and **(j)** indicate the molecular mass (Kd).

Fig. s8. Original Western blots, parts of which are shown in Fig. 5 The gel shown in **(a)** (from left to right) was loaded with the prestained protein ladder (Thermo Scientific) and lysates respectively prepared from ARC tissues of rats without any treatment (Naïve) and rats which received the intra-ARC infusion of sh-NC (sh-NC, middle) or sh-Src (sh-Src, right) but no CFA injection (No CFA). The group of blots in **(a)** was stripped and successively probed with an antibody against GluN2B-pY1472 (2B-pY1472, top blot), GluN2B (middle blot), or GAPDH (bottom blot). The gels shown in **(b)** – **(e)** were loaded (from left to right) with the prestained protein ladder (Thermo Scientific) and lysates respectively prepared from ARC tissues of naïve rats without any treatment in the same experimental sets and rats [which received the intra-ARC infusion of sh-NC (CFA/sh-NC) or sh-Src (CFA/sh-Src) 7 days before] at day 1 to day 14 after the CFA injection as indicated. The groups of blots shown respectively in **(b)** – **(e)** were stripped and successively probed with an antibody against GluN2B-pY1472 (2B-pY1472, top blot), GluN2B (middle blot), or GAPDH (bottom blot). Values on the right side of blots shown in **(e)** indicate the molecular mass (Kd).

Src activation in the hypothalamic arcuate nucleus may play an important role in pain hypersensitivity

Hanpei Ma§, Chunxu Yao§, Peng Ma, Ju Zhou, Shan Gong, Jin Tao, Xian-Min Yu*, Xinghong Jiang*

Key Laboratory of Pain Basic Research and Clinical Therapy, Department of Physiology and Neurobiology, Medical College of Soochow University, Suzhou 215123, China

§: Contributed equally

* Corresponding authors:

yuxianminyu1981@hotmail.com; jiangxinghong@suda.edu.cn

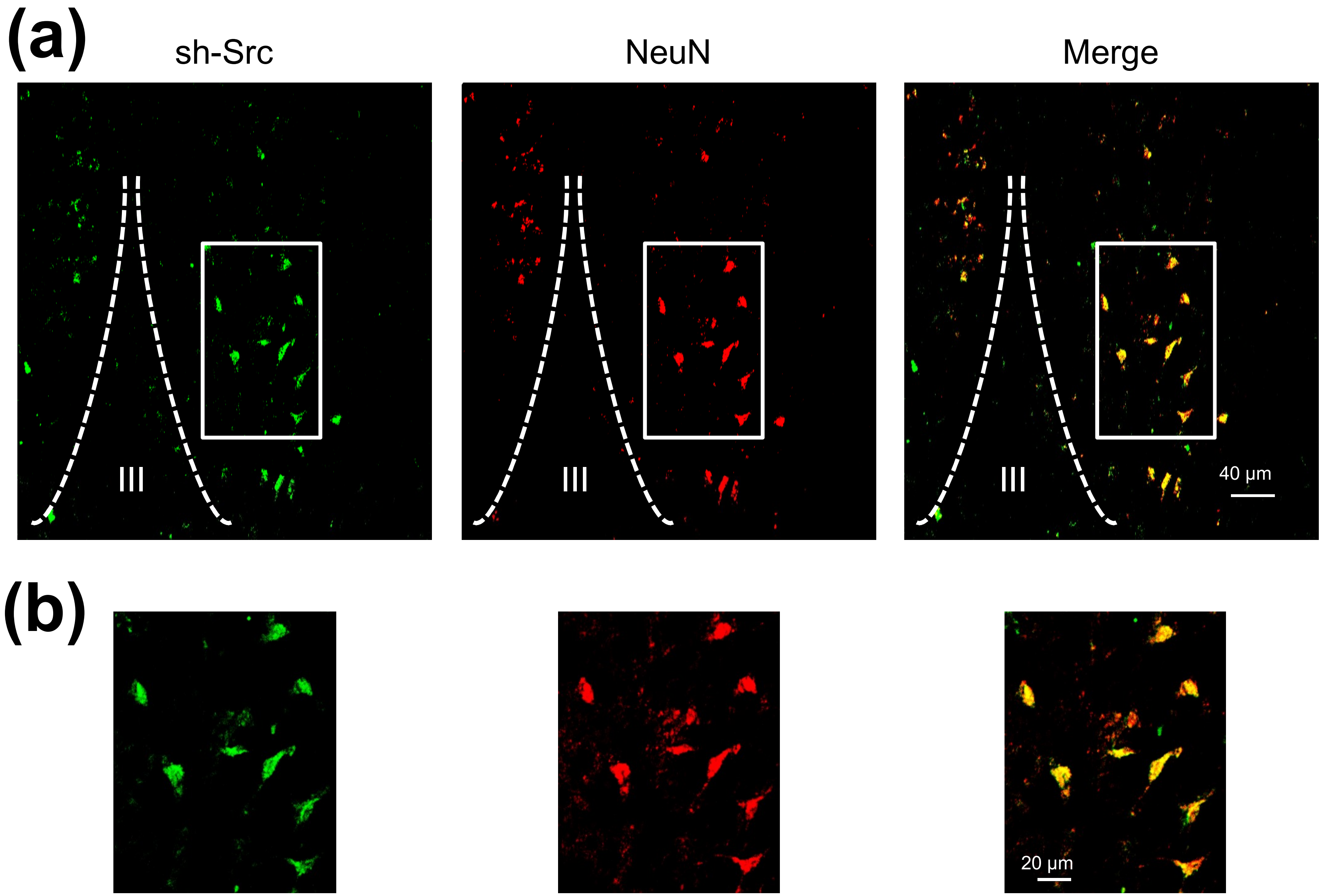

Ma et al Fig. s1

Src activation in the hypothalamic arcuate nucleus may play an important role in pain hypersensitivity

Hanpei Ma[§], Chunxu Yao[§], Peng Ma, Ju Zhou, Shan Gong, Jin Tao, Xian-Min Yu[*], Xinghong Jiang[*]

Key Laboratory of Pain Basic Research and Clinical Therapy, Department of Physiology and Neurobiology, Medical College of Soochow University, Suzhou 215123, China

[§]: Contributed equally

* Corresponding authors:

yuxianminyu1981@hotmail.com; jiangxinghong@suda.edu.cn

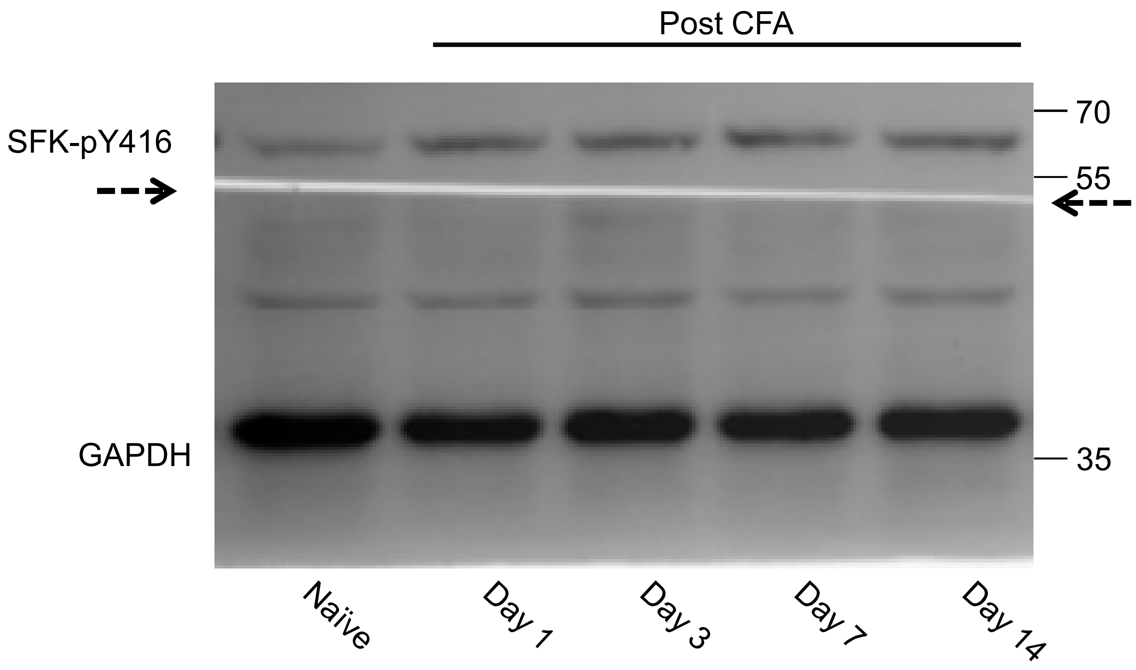
Post CFA
SFK-pY416
GAPDH
70
55
35
Naïve
Day 1
Day 3
Day 7
Day 14

Src activation in the hypothalamic arcuate nucleus may play an important role in pain hypersensitivity

Hanpei Ma[§], Chunxu Yao[§], Peng Ma, Ju Zhou, Shan Gong, Jin Tao, Xian-Min Yu[*], Xinghong Jiang[*]

Key Laboratory of Pain Basic Research and Clinical Therapy, Department of Physiology and Neurobiology, Medical College of Soochow University, Suzhou 215123, China

[§]: Contributed equally

* Corresponding authors:

yuxianminyu1981@hotmail.com; jiangxinghong@suda.edu.cn

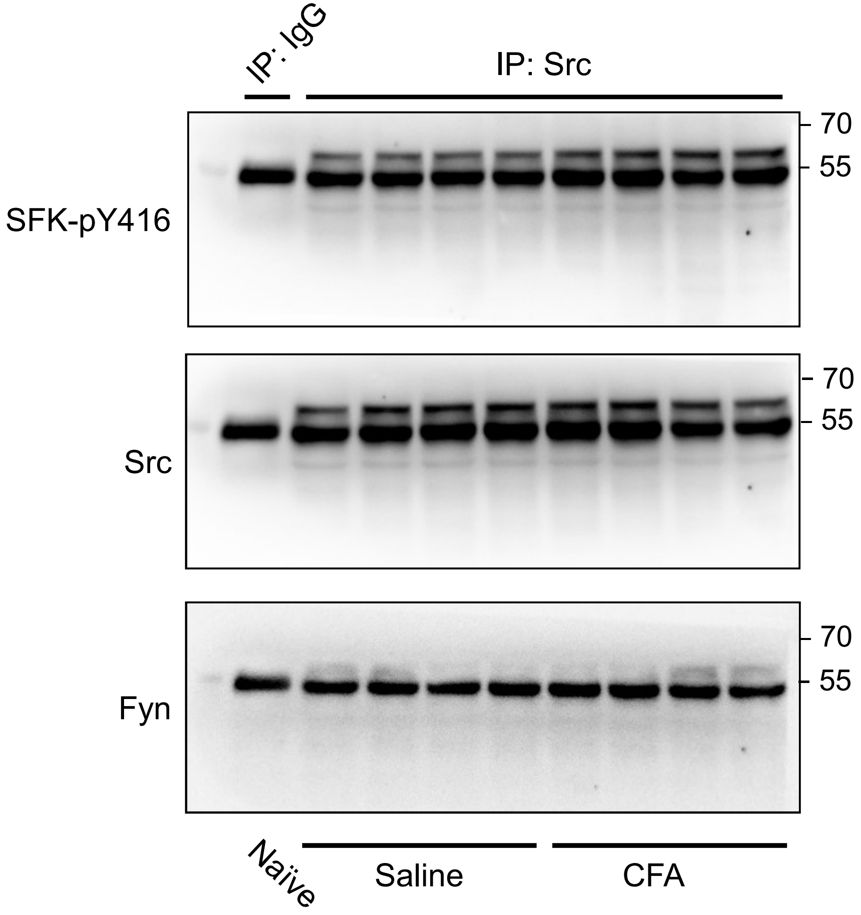
IP: IgG
IP: Src
SFK-pY416
Src
Fyn
70
55
70
55
70
55
Naïve
Saline
CFA

Src activation in the hypothalamic arcuate nucleus may play an important role in pain hypersensitivity

Hanpei Ma[§], Chunxu Yao[§], Peng Ma, Ju Zhou, Shan Gong, Jin Tao, Xian-Min Yu[*], Xinghong Jiang[*]

Key Laboratory of Pain Basic Research and Clinical Therapy, Department of Physiology and Neurobiology, Medical College of Soochow University, Suzhou 215123, China

[§]: Contributed equally

* Corresponding authors:

yuxianminyu1981@hotmail.com; jiangxinghong@suda.edu.cn

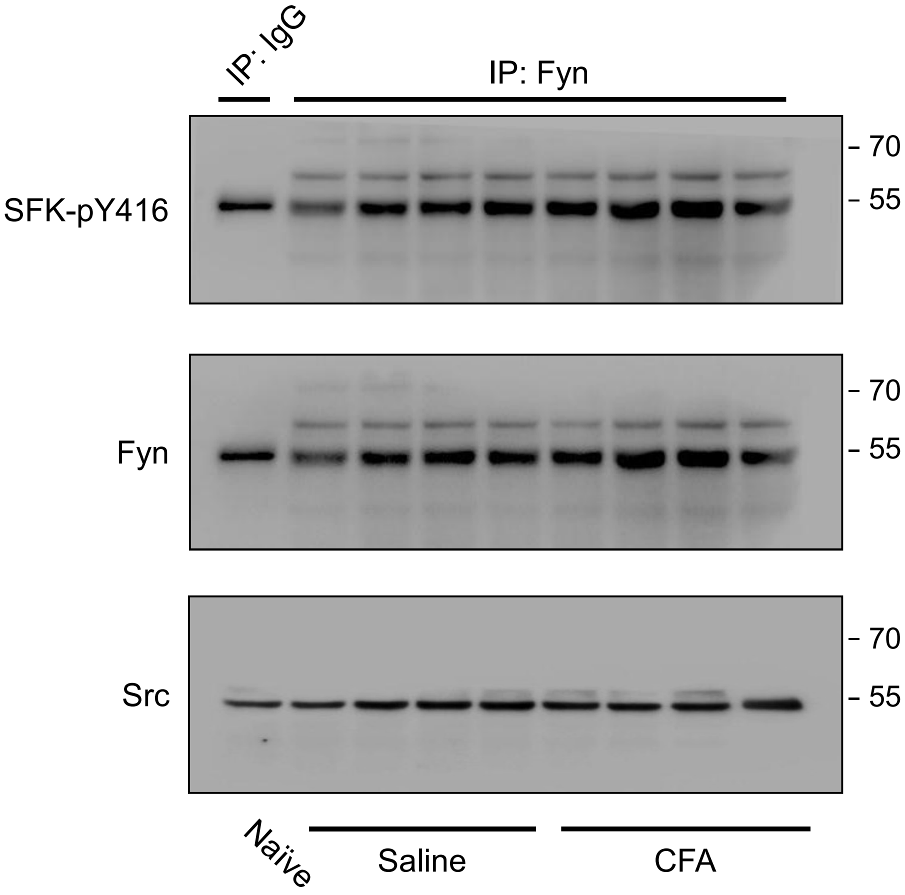
IP: IgG
IP: Fyn
SFK-pY416
Fyn
Src
70
55
70
55
70
55
Naïve
Saline
CFA

Src activation in the hypothalamic arcuate nucleus may play an important role in pain hypersensitivity

Hanpei Ma[§], Chunxu Yao[§], Peng Ma, Ju Zhou, Shan Gong, Jin Tao, Xian-Min Yu[*], Xinghong Jiang[*]

Key Laboratory of Pain Basic Research and Clinical Therapy, Department of Physiology and Neurobiology, Medical College of Soochow University, Suzhou 215123, China

[§]: Contributed equally

* Corresponding authors:

yuxianminyu1981@hotmail.com; jiangxinghong@suda.edu.cn

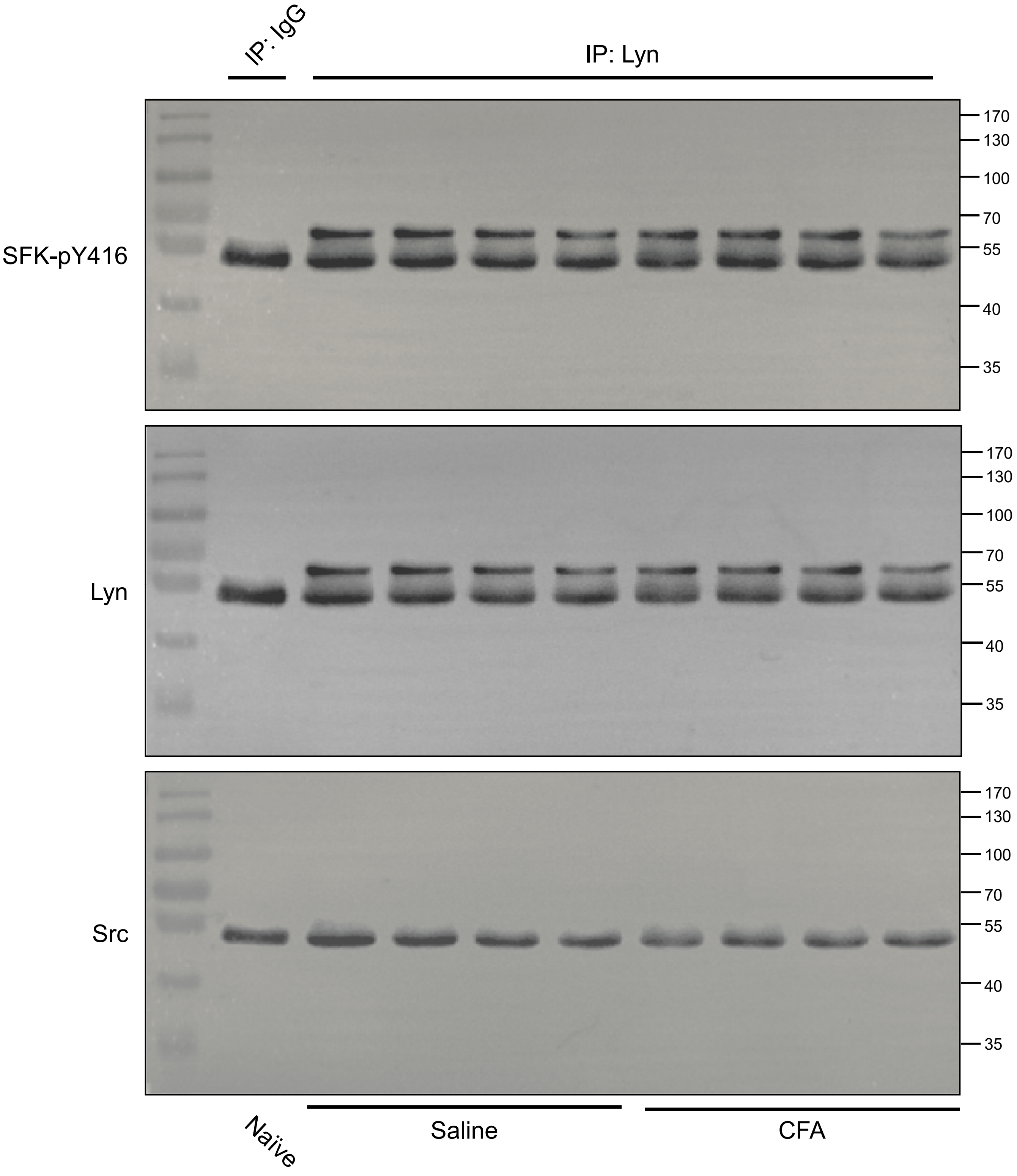
IP: IgG
IP: Lyn
SFK-pY416
Lyn
Src
170
130
100
70
55
40
35
Naïve
Saline
CFA

Src activation in the hypothalamic arcuate nucleus may play an important role in pain hypersensitivity

Hanpei Ma[§], Chunxu Yao[§], Peng Ma, Ju Zhou, Shan Gong, Jin Tao, Xian-Min Yu[*], Xinghong Jiang[*]

Key Laboratory of Pain Basic Research and Clinical Therapy, Department of Physiology and Neurobiology, Medical College of Soochow University, Suzhou 215123, China

[§]: Contributed equally

* Corresponding authors:

yuxianminyu1981@hotmail.com; jiangxinghong@suda.edu.cn

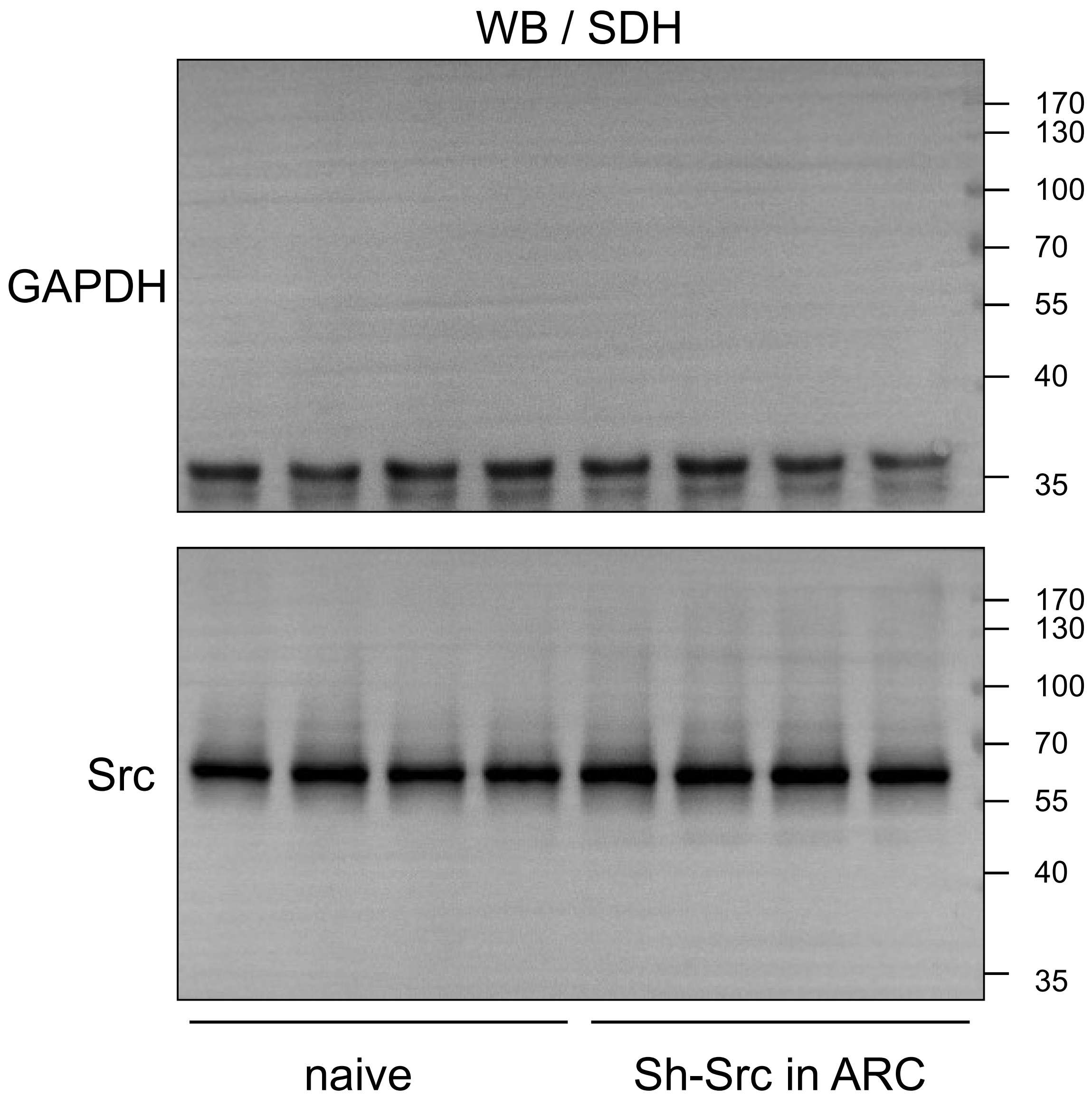
WB / SDH
GAPDH
Src
170
130
100
70
55
40
35
170
130
100
70
55
40
35
naive
Sh-Src in ARC

Src activation in the hypothalamic arcuate nucleus may play an important role in pain hypersensitivity

Hanpei Ma[§], Chunxu Yao[§], Peng Ma, Ju Zhou, Shan Gong, Jin Tao, Xian-Min Yu[*], Xinghong Jiang[*]

Key Laboratory of Pain Basic Research and Clinical Therapy, Department of Physiology and Neurobiology, Medical College of Soochow University, Suzhou 215123, China

[§]: Contributed equally

* Corresponding authors:

yuxianminyu1981@hotmail.com; jiangxinghong@suda.edu.cn

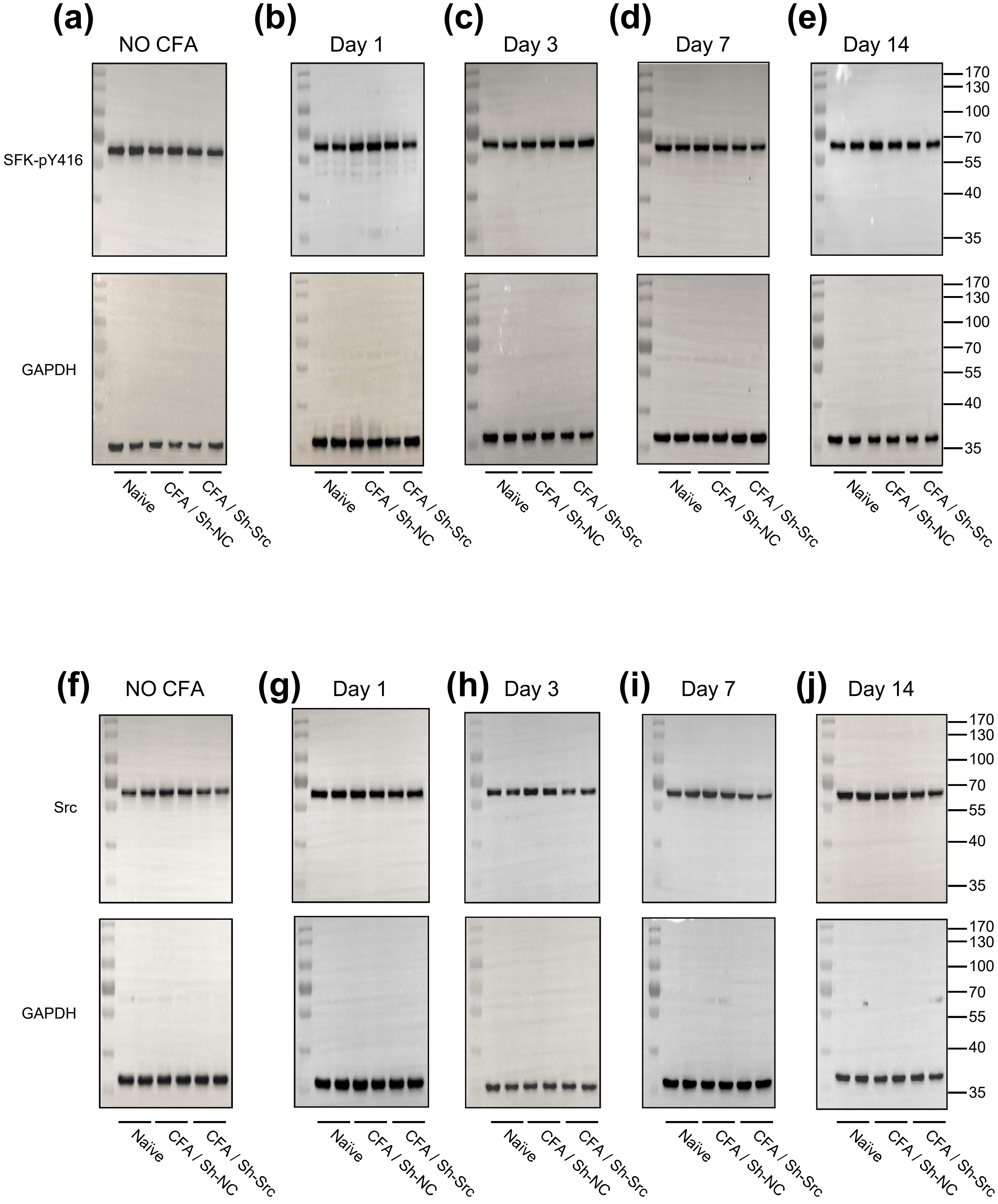

(a) NO CFA
(b) Day 1
(c) Day 3
(d) Day 7
(e) Day 14
SFK-pY416
GAPDH
170
130
100
70
55
40
35
Naïve
CFA / Sh-NC
CFA / Sh-Src
(f) NO CFA
(g) Day 1
(h) Day 3
(i) Day 7
(j) Day 14
Src
GAPDH

Src activation in the hypothalamic arcuate nucleus may play an important role in pain hypersensitivity

Hanpei Ma[§], Chunxu Yao[§], Peng Ma, Ju Zhou, Shan Gong, Jin Tao, Xian-Min Yu[*], Xinghong Jiang[*]

Key Laboratory of Pain Basic Research and Clinical Therapy, Department of Physiology and Neurobiology, Medical College of Soochow University, Suzhou 215123, China

[§]: Contributed equally

* Corresponding authors:

yuxianminyu1981@hotmail.com; jiangxinghong@suda.edu.cn

Ma et al. Fig. s8

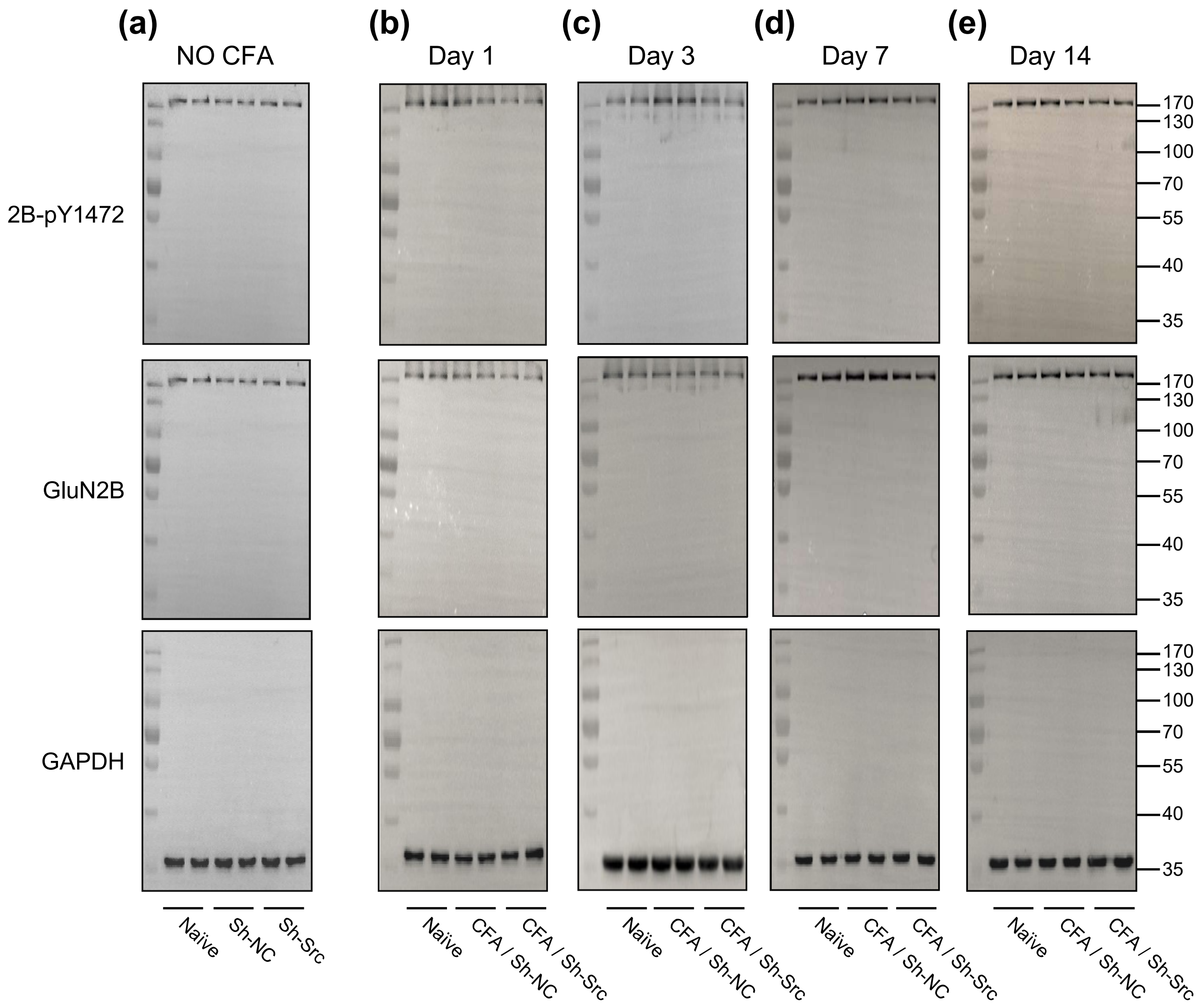

Supplement: Supplementary file 1 — Supplementary Figures [file 41598_2019_40572_MOESM1_ESM.pdf]
